# Treatment Assessment of pNET and NELM after Everolimus by Quantitative MRI Parameters

**DOI:** 10.3390/biomedicines10102618

**Published:** 2022-10-18

**Authors:** Maria Ingenerf, Sophia Kiesl, Michael Winkelmann, Christoph J. Auernhammer, Johannes Rübenthaler, Freba Grawe, Matthias P. Fabritius, Jens Ricke, Christine Schmid-Tannwald

**Affiliations:** 1 Department of Radiology, University Hospital, LMU Munich, 81377 Munich, Germany; 2 ENETS Centre of Excellence, Interdisciplinary Center of Neuroendocrine Tumours of the GastroEnteroPancreatic System at The University Hospital of Munich (GEPNET-KUM), 81377 Munich, Germany; 3 Department of Internal Medicine 4, University Hospital, LMU Munich, 81377 Munich, Germany

**Keywords:** therapy response, neuroendocrine tumors, DWI, everolimus

## Abstract

Assessment of treatment response to targeted therapies such as everolimus is difficult, especially in slow-growing tumors such as NETs. In this retrospective study, 17 patients with pancreatic neuroendocrine tumors (pNETs) and hepatic metastases (NELMs) (42 target lesions) who received everolimus were analyzed. Intralesional signal intensities (SI) of non-contrast T1w, T2w and DCE imaging, and apparent diffusion coefficients (ADCmean and ADCmin) of DWI, were measured on baseline and first follow-up MRI after everolimus initiation. Response assessment was categorized according to progression-free survival (PFS), with responders (R) showing a PFS of ≥11 months. ADCmin of NELMs decreased in Rs whereas it increased in non-responders (NR). Percentual changes of ADCmin and ADCmean differed significantly between response groups (*p* < 0.03). By contrast, ADC of the pNETs tended to increase in Rs, while there was no change in NRs. Tumor-to-liver (T/L) ratio of T1 SI of NELMs increased in Rs and decreased in NRs, and percentual changes differed significantly between response groups (*p* < 0.02). T1 SI of the pNETs tended to decrease in Rs and increase in Ns. The quotient of pretherapeutic and posttherapeutic ADCmin values (DADCmin) and length of everolimus treatment showed significant association with PFS in univariable Cox analysis. In conclusion, quantitative MRI, especially DWI, seems to allow treatment assessment of pNETs with NELMs under everolimus. Interestingly, the responding NELMs showed decreasing ADC values, and there might be an opposite effect on ADC and T1 SI between NELMs and pNETs.

## 1. Introduction

Neuroendocrine tumors of the pancreas (pNETs) represent a very rare tumor entity, with an incidence of 0.48 per 100.000 population and account for only 1% of all pancreatic tumors [1,2,3]. Due to the slow growth and the frequently unspecific symptoms—especially in the case of non-functional-pNETs—tumor stage is often advanced at the time of diagnosis, with hepatic metastases in around 64–70% of cases [4] Thus, curative tumor resection is limited, and therapy concepts are based on symptom control and reduction of tumor spread [5].

One of the most promising new therapy strategies for advanced NETs is based on the targeting and inhibition of the mTOR protein [6]. The mTOR pathway plays an important role in the tumorigenesis of NETs of different origins [1,6]. Due to its antiproliferative and antiangiogenic effects, everolimus, an oral inhibitor of the mTOR pathway, nowadays represents an integral option in the second-line therapy for progressive disease under SSAs or cytotoxic chemotherapy [7,8].

The RADIANT-3 trial proved the efficacy of everolimus in advanced pancreatic NETs, prolonging median PFS (11 months vs. 4.6 months for placebo group) and improving overall survival (OS) by 6.3 months (44 vs. 37 months) [1,9,10]. However, objective tumor response rates were generally low (5% in the everolimus group), indicating that the positive effect on PFS was mainly due to stabilization of tumor growth or minor tumor shrinkage, not reaching the cut-off for partial response as defined by RECIST [9,11]. An increasing body of evidence suggests that conventional response criteria, which are mainly based on tumor size change, are suboptimal to evaluate treatment response to antiproliferative or antiangiogenic effects as mediated by targeted anti-cancer drugs, especially in slow-growing tumors such as NETs [11,12,13]. A recently published study showed that the Choi criteria, which integrate changes in tumor density in addition to size, correlated better with OS than RECIST in the therapy evaluation of pNETs under sunitinib [14]. Functional imaging parameters, especially diffusion-weighted imaging (DWI), provide encouraging capabilities to serve as new imaging biomarkers [15,16,17,18,19,20,21]. The European Neuroendocrine Tumor Society (ENETS) has already discussed ADC as a promising (despite little evidence so far) predictive parameter for tumor response in its expert consensus guideline and recommended the more frequent use of ADC in addition to RECIST [22]. Studies evaluating morphological response criteria of patients with NETs receiving everolimus or sunitinib are largely missing. However, this would be of fundamental importance, to allow an early differentiation of responders from non-responders in clinical practice and improve oncological study design by a more accurate definition of PFS. 

Therefore, the purpose of this study was to analyze the diagnostic reliability of quantitative MRI for therapy response assessment in pNETs under everolimus. 

## 2. Materials and Methods

### 2.1. Patients

**Figure 1:**
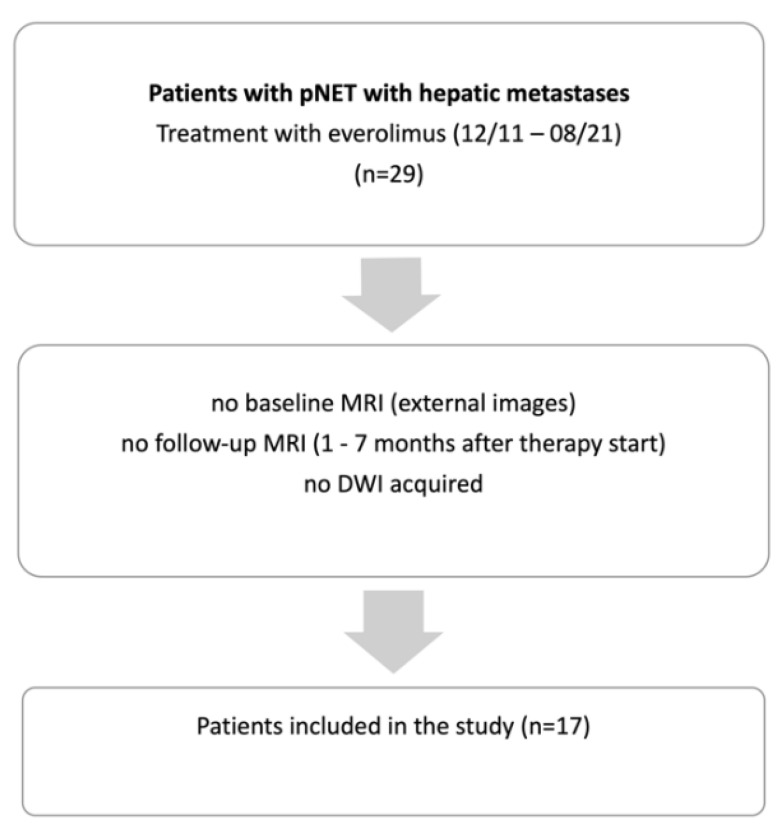
Flow-chart illustrating retrospective patient selection.

Consecutive patients with histologically proven pNETs (resected or advanced) who received everolimus at our department, with therapy start between December 2011 and August 2021, were retrospectively included in this study. Further inclusion criteria were primary or secondary liver metastases and a baseline and follow-up MRI with DWI (Figure 1). Patient selection for therapy with everolimus was based on a consensus decision in an interdisciplinary tumor conference certified for NETs (ENETS Center of Excellence). The local research ethics committee (LMU Munich, project number 20-0847) approved this retrospective study and the need for written informed patient consent was waived.

### 2.2. MR Imaging

MR examinations were obtained using a 1.5T and 3T scanner (Magnetom Avanto, Magnetom Aera, Magnetom Skyra and Magnetom Verio, Siemens Healthcare, Erlangen, Germany). The imaging protocol consisted of an unenhanced T1w gradient-echo (GRE) sequence in- and out-of-phase, a single shot T2w sequence T1w 3D GRE sequences with fat suppression (fs) (non-contrast and 20, 50, 120 s after intravenous contrast media injection (Gd-EOB-DTPA; Primovist, Eovist, Bayer Schering Pharma, Germany; 25 µmol/kg body weight) depending on circulation time), a multishot T2w turbo spin echo sequence with fat saturation, diffusion-weighted sequences (b-values: 50, 400, 800 s/mm^2^) and a T1w GRE sequence with fat saturation and a T1w 3D GRE fs sequence after a delay of 15 min. ADC maps were generated from DW images, including all b-values.

### 2.3. Image Analysis

All pretherapeutic MRI data were reviewed by two radiologists in consensus (C.S. and S.K., with 15 and 2 years of experience in abdominal MRI). In consensus, they defined two hepatic metastases (minimum size > 0.5 cm) per patient as target lesions and assessed if the pNET was resected. Inclusion criteria for metastases were homogenous appearance and no artifacts within the lesion in all sequences. Quantitative MRI evaluation was conducted in two separate sessions by two readers independently: (1) preinterventional MRI and (2) postinterventional MRI with a 2-week interval between the review sessions. The readers were blinded for the patients’ clinical or follow-up data. ADC values were calculated by manually placing a circular region-of-interest (ROI) on the slice with the largest tumor extent on DWI (excluding structures close to the rim to avoid partial volume effects) and on non-enhanced T2w and T1w + contrast-enhanced images (arterial, portal-venous, venous, and hepatobiliary phase), SI values were recorded by drawing ROIs of the lesions as large as possible. In addition, ADC, T2w and T1w SI values of normal liver, pancreas and spleen were measured on pre- and posttherapeutic scans, by placing circular ROIs, as large as possible, in areas of tumor-free tissue. The size (longest diameter) of hepatic lesions was measured on T1-weighted hepatobiliary phase and on T1w arterial phase for the pNETs. 

### 2.4. Standard of Reference and Response to Treatment

For all patients in the study cohort, diagnosis of pNET was confirmed by histopathology, and for most patients Ki-67 labelling index of the primary tumor was obtained. Grading was performed according to the 2017 WHO Tumor Classification Guideline (G1: Ki-67 Index was <3%, G2: Ki-67 Index was 3–20%, and G3 NET/NEC: Ki-67 Index was >20%) [23]. Treatment response was evaluated as progression-free survival (PFS) and was calculated in months from the time of everolimus initiation until progression according to the local interdisciplinary tumor board, including evaluation of all performed imaging studies (CT, PET/CT, MRI). Responders were defined by PFS ≥ 11 months and non-responders (NRs) by PFS < 11 months, respectively. This cut-off was based on the reported median PFS of 11 months in the RADIANT-3 phase-III-trial. Additionally, OS was determined in months from treatment start until death from any cause. Patients who were still alive at the time of last follow-up (May 2022) were censored. 

### 2.5. Statistical Analysis

Statistical analysis was performed utilizing commercially available statistical software (Graphpad Prism Version 6, San Diego, Calif. and SPSS version 25, Chicago, IL, USA). Statistical significance level was defined at *p* ≤ 0.05. All data was expressed as mean or median values with standard deviation (SD) or interquartile range [IQR], respectively. Normal distribution of continuous variables was evaluated by visual inspection of the frequency distribution (histogram). We compared the SI and ADC values of target lesions before and after therapy using a two-tailed, paired t-test. Two-tailed, unpaired t-tests were used to compare the SI and ADC values of target lesions between different response groups, respectively. The Wilcoxon rank-sum test was used in case of a non-normal distribution. OS and PFS were modeled according to the Kaplan–Meier method. The multivariable Cox proportional hazards regression model was performed to analyze independent prognostic clinical and imaging parameters for PFS and OS. Using a stepwise approach, all variables with *p* < 0.1 in univariable analysis were entered into the multivariable model.

## 3. Results

### 3.1. Patients

Seventeen patients (6 female, 11 male) with a total of 42 target lesions (34 liver metastases, 8 primary tumors) were included in this retrospective study. Due to motion artifacts, DWI was not evaluated in one patient and two pNETs were not evaluated due to intratumoral calcifications and heterogenous appearance, respectively. Baseline MRI was acquired 26 ± 32 d prior to treatment start, and first follow-up MRI was performed 115 ± 44 d after initiation of everolimus. A total of 7/17 patients underwent primary tumor resection. Most of the included NET patients had G2 tumors (n = 14), followed by two high-grade tumors (G3), while no low-grade (G1) tumors were included, and for one patient no grading was obtained. Detailed patient characteristics are presented in Table 1. 

### 3.2. Overall Survival and Progression-Free Survival

By the end of the study, 9/17 patients had died (53%), and 15 (88%) had shown progression on imaging. Overall median PFS was 5 months (95% CI: 3.5–6.6). Median PFS was 31 months (95% CI: 13.8–48.2) in the R group (n = 5), and 3 months in the NR group (95% CI: 2.5–3.5). Overall median survival was 36 months (95% confidence interval (CI): 5.5–66.5), with a median follow-up time of 38 months. Median OS in Rs was 38 months (95% CI: 0–87) compared to 21 months (95% CI: 4.3–37.8) in NRs. Survival and PFS of patients with elevated vs. non-elevated baseline CgA and NSE levels did not differ significantly (*p* > 0.67). 

### 3.3. ADC Measurements

**Figure 2:**
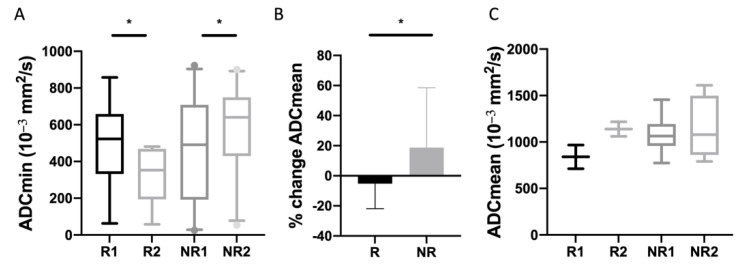
Analysis of ADC changes in NELMs and pNETs. (**A**) ADCmin of NELMs on baseline and first FU imaging. (**B**) Percentual changes of ADCmean of NELMs. (**C**) ADCmean of pNETs on baseline and first FU imaging. Asterisks indicate statistical significance (* *p* ≤ 0.05).

**Figure 3:**
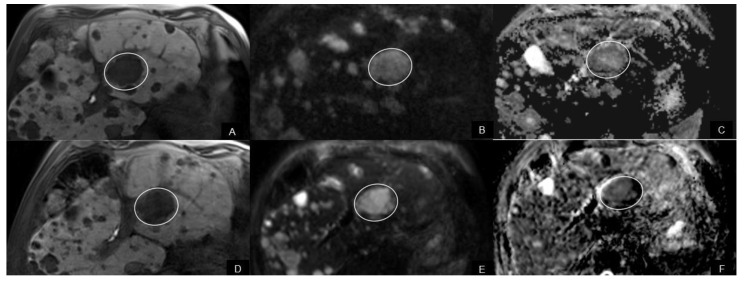
A 66-year-old male with liver metastases of pNET classified as a responder. (**A**) The pre-interventional axial T1w image (hepatobiliary phase) shows hypointense metastasis (circle) in segment 2. The metastasis (arrow) shows restricted diffusion with high signal on axial DW-MR image b = 800 s/mm^2^ (**B**,**D**) dark signal (circle) on ADC map (**C**). The pretherapeutic ADCmin value of the metastasis was 858 × 10^−3^ mm^2^/s. Under everolimus, the metastasis showed no significant change in size on hepatobiliary phase (**D**), however there was a loss of signal on DW-MR image b = 800 s/mm^2^ (**E**) compared to the pre-interventional image and decreasing signal (circle) on the ADC map (**F**). The post-interventional ADCmin values were 476 × 10^−3^ mm^2^/s and lower higher compared to pretherapeutic ADC value.

**Figure 4:**
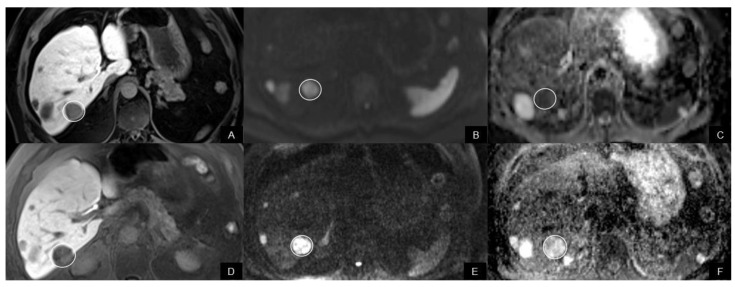
An 74-year-old man with hepatic metastasis of pNET classified as a nonresponder. (**A**,**D**) Under everolimus, the metastasis (arrow) increased in size from (**A**) 25 mm to (**D**) 29 mm on the axial contrast-enhanced T1W image (liver-specific phase) (**B**,**D**). On both (**B**) pre- and (**D**) posttherapeutic MRI, the metastasis (arrow) showed restricted diffusion with high signal on DWI image with high b-value. (**C**) ADCmin value of the metastasis (circle) on pretherapeutic ADC map (**E**) was 747 × 10^−3^ mm^2^/s and increased to 811 × 10^−3^ mm^2^/s on (**F**) the post-therapeutic scan.

There were no significant differences of ADC values of NELMs between Rs and NRs before treatment. ADCmin in responding NELMs decreased significantly after therapy (*p* < 0.02), while ADCmin increased in non-responding NELMs (*p* < 0.03) (Figure 2A, Figure 3 and Figure 4) Concordantly, percentual changes of ADCmin in NELMs were significantly different between response groups (*p* = 0.001) with a median decrease of −40.5% (IQR −48.9—−4.1) in Rs compared to an increase of 28.7% (IQR −4.6–160.1) in NRs. Additionally, for ADCmean, there was a significant increase in first FU in NRs (*p* < 0.02), while there was no significant change in Rs. Percentual changes of ADCmean differed significantly between response groups (*p* = 0.03), with a decrease in Rs (−5.2%, IQR −21.9—5.5) compared to an increase in NRs (18.7%, IQR −5.6–58.6) (Figure 2B). 

Regarding the primary tumor, statistical analysis was limited due to the small sample size. In Rs, both ADCmin and ADCmean tended to increase after therapy initiation, while there was no change in NRs (Figure 2C). For ADCmean, percentual change was 37.1% (IQR 25.6–48.5) in Rs compared to 1.2% (IQR −13.4–25) in NRs. 

There were no significant changes between the pre- and posttherapeutic ADCmean of non-tumorous liver, spleen, and pancreatic tissue.

### 3.4. T2 Signal

**Figure 5:**
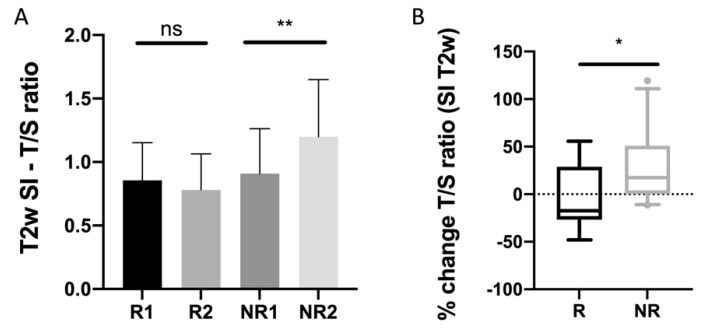
(**A**) T/S ratio of non-enhanced T2w SI of NELMs before and after treatment start. (**B**) Percentual changes of T/S ratio of NELMs. Asterisks indicate statistical significance (* *p* ≤ 0.05, ** *p* ≤ 0.01).

T2w of NELM divided by the T2w background signal of the spleen (T/S ratio) increased significantly in NRs while there was no change in Rs (Figure 5). 

### 3.5. Non-Enhanced T1 Signal

**Figure 6:**
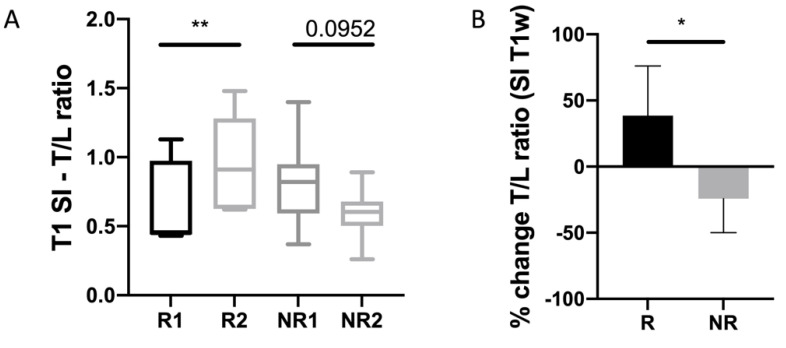
(**A**) T/L ratio of non-enhanced T1w of NELM on baseline and first FU. (**B**) Percentual change of T/L ratio. Asterisks indicate statistical significance (* *p* ≤ 0.05, ** *p* ≤ 0.01).

The ratio of T1 SI of the target lesion divided by T1 SI of the liver (T/L ratio) showed a significant increase in the R group after therapy start, while T/L ratio tended to decrease in the NR group (Figure 6A). Concordantly, percentual changes of non-enhanced T/L ratio differed significantly between response groups (*p* = 0.02) with an increase of 38.6% in the R group (IQR 31.8–76.1), compared to a decrease of −24.2% in the NR group (IQR −50–9.9) (Figure 6B). Non-enhanced T1 of the pNET tended to decrease in Rs and slightly increase in NRs; however, statistical analysis was not possible due to the small number of included pNETs. Native TI SI of non-tumorous liver and pancreas did not differ significantly between groups, and after the start of everolimus. 

### 3.6. DCE

**Figure 7:**
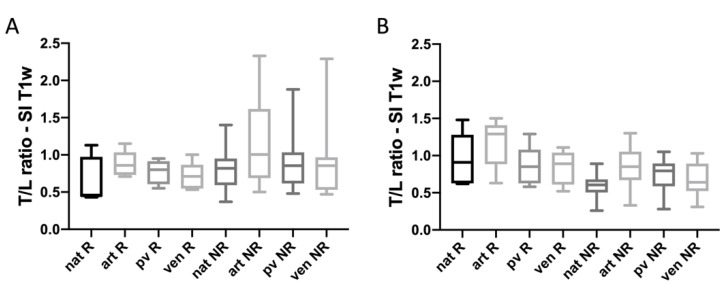
Analysis of T/L ratio of SI of T1w on non-enhanced, arterial, portal-venous and venous phase for responding NELMs (PFS > 11 m) and non-responding NELMs (PFS < 11 m) on pretreatment images (**A**) and first FU MRI (**B**).

There were no significant differences in the contrast enhancement of NELM between arterial, pv and venous phases between response groups. However, in accordance with the results of non-enhanced T1w SI evaluation, background T1 T/L ratio after therapy start tended to have higher SI ratios in responding lesions than non-responding NELMs. For arterial phase, T/L ratio was 1.18 in Rs compared to 0.86 in NRs (*p* < 0.05) (Figure 7). 

### 3.7. Cox Regression of PFS and OS

In univariable analysis of PFS, length of everolimus treatment and the imaging parameter DADCmin, which represents the quotient of ADCmin of NELM before and after therapy start (ADCmin pretherapeutic/ADCmin posttherapeutic = DADCmin), showed a significant association with PFS. The other clinical parameters, including age, ki-67, grading and elevated baseline NSE or Chromogranin A levels showed no significant associations with PFS in the univariable analysis (Table 2). In the multivariable model, neither of both parameters remained significant; however, the *p*-value of DADCmin was borderline (0.09), especially regarding the small cohort for Cox analysis. In the univariable analysis for OS, none of the parameters showed significant association with patient outcome (Table 3). 

## 4. Discussion

In this study, we found that quantitative MRI parameters, especially ADC and T1w SI of NELMs, were useful for response assessment of pNETs treated by the targeted agent everolismus. We chose PFS as the primary end point, as recommended by the National Cancer Institute Neuroendocrine Tumor Clinical Trials Planning Meeting consensus report. The use of OS is especially challenging in NETs due to generally long survival times, and consequently a wider range of post-progression salvage therapies [4]. Overall median PFS was rather low in our study cohort, with 5 months compared to a median PFS of 11 months in the RADIANT-3 trial [9]. Overall median survival was also slightly lower than reported, with 36 months in our study compared to 44 months in the RADIANT-3 trial [4]. Factors which might cause this difference in our small study cohort in comparison to the RADIANT-3 trial data might be tumor grading, percentage of patients with high baseline NSE 1 × ULN or high baseline CgA 2 × ULN, as well as late treatment line with everolimus. In the RADIANT-3 trial, 82% had well-differentiated tumors and only 17% moderately differentiated tumors, while in our cohort 82% had moderately differentiated tumors and 12% low-differentiated tumors. In the RADIANT-3 trial chromogranin, A was elevated in 46% and NSE was elevated in 26% of all patients, while in our patient group CgA was elevated in 76% and NSE was elevated in 53% [24,25,26]. 

Interestingly, we found that ADCmin values showed a significant decrease after everolimus initiation in responding NELMs, while ADC in NRs increased. The quotient of ADCmin pretherapeutic/ADCmin posttherapeutic was the only imaging parameter that showed a significant association with PFS in univariable Cox analysis (HR 0.21 (95% CI 0.06–0.8; *p* = 0.02). This underlines that lower ADCmin values in follow-up MRIs were associated with better treatment response. This finding seems counterintuitive at first sight, as therapies inducing apoptosis usually show an increase in ADC values due to tumor necrosis and lysis; however, it depends on the mechanism of treatment. For example, PRRT in neuroendocrine tumors has been reported to cause increased ADC values in patients with better OS and PFS [16], and the percentual increase of ADC was found to correlate with decrease in size [18].

The effect of targeted, cytostatic agents is not immediate cell death as in cytotoxic regimens. pNETs characteristically contain a high microvascular density, which is expressed by radiological enhancement [14]. Previous studies investigating targeted therapies with antiangiogenic effects have shown (a transient) decrease in tumor ADC values, which might be accompanied by reduced contrast enhancement. 

It is likely that antiangiogenic effects reduce vascular permeability, which is accompanied by lower perfusion and extravascular–extracellular space, and thus decreasing ADC. Only larger tumor necrosis mediated by vascular-directed therapies would lead to an increase of ADC [11,27]. 

Schraml et al. investigated early changes of DWI in advanced hepatocellular carcinoma treated with the multikinase inhibitor sorafenib and found an early decrease in ADC, followed by a later re-increase. The authors suggested that these changes were caused by hemorrhagic tumor necrosis. Antiangiogenic treatments induce ischemic conditions, which causes a disruption of the cell membrane and in turn leads to a shift of extracellular ions and water molecules into the cell while reducing the extracellular volume [11,28,29].

Additionally, for patients who received anti-VEGF drugs in brain tumors, a transient decrease of ADC was reported [30,31]. Patients with renal cell carcinoma, which is also a highly vascular tumor, treated with sunitinib showed an early reduction of D (IVIM) of the primary tumor, with a larger reduction being associated with a better RECIST response and longer PFS [32]. It was shown that in spite of increased histological necrosis, the radiological-assessed diffusion restriction increased [33]. 

Another influencing factor could be related to the immunological effects, as everolimus is known to increase the rate of regulatory T-cells [34]. Additionally, increased fibrosis due to replacement of viable tumor cells by collagen was hypothesized as a cause for decreasing ADC values in responding hepatic metastases indicated by improved OS [35]. 

For evaluation of T1 SI acquisition parameters are not standardized and studies evaluating comparability are missing. Native T1 SI of NELM decreased in NR and increased in R—both for absolute values as well as for the relative ratio of T1 SI of the target lesion divided by T1 SI of the liver (T/L ratio). NELM in the R group showed an increase of around 39% after everolimus initiation (vs. −24% for NR). Two different studies, both analyzing HCC, found hyperintense SI of non-enhanced T1w after treatment with sorafenib [28,36], suggesting that therapy induced intralesional hemorrhage or protein-rich necrosis. Mostly, no intratumoral (macro-) hemorrhage was detected in T1w images in our study cohort, but this might be explained by microbleeding, which is not visually noticeable but affects T1w SI and DWI, and according to the time-dependent evolution of blood products, the hemoglobin might be obscured by the fat saturation [28]. 

Elevated baseline levels of neuron-specific enolase (NSE) and chromogranin A (CgA) were shown to be prognostic for poor survival in pNETs under everolimus [1,37]. In our study cohort, DADCmin correlated better with OS than baseline CgA, while there was no significant correlation with pretherapeutic NSE levels, which might be explained by a small sample size. 

Tumor angiogenesis is supposed to play a crucial role in response assessment of targeted therapies [12]. Regarding the quantitative DCE measurements, we found no significant changes in tumor enhancement. However, the analysis of enhancement is prone to errors as it depends on the exact timing of contrast-enhanced images, and different models for tracer kinetics are used by different research groups. In this study, DCE (including four contrast phases) was used instead of a high-resolution over-time perfusion technique. In addition, quantitative DCE evaluation, e.g., quantitative EASL (European Association for the Study of Liver) is still labor- and time-intense, and thus not suitable for clinical routine [38]. 

Evaluation of ADC and non-enhanced SI of T1w of the pNETs tended to show opposite effects compared to the NELM. T1w SI of the pNET tended to decrease in R and slightly increase in NR, and ADC (min and mean) of the pNETs tended to increase in R, while there was no change in NR. However, statistical analysis was not possible due to the small number of included pNETs, as they were either resected (n = 7) or strong intratumoral heterogeneities impeded quantitative evaluation. Thus, results of the quantitative evaluation of pNETs should not be overinterpreted; however, we included the data for possible future systematic reviews. 

One major limitation of this study is the small sample size, especially of pNETs, due to the low incidence of pNETs and everolimus representing a second-line treatment. As the study design was retrospective, time intervals were not entirely homogenous and imaging data were not acquired on a single MRI scanner. Therefore, for T1w and T2w SI measurement, the ratio of the background liver/spleen was calculated to eliminate scanner-dependent confounding factors. DWI represents simultaneous information regarding diffusion and microperfusion, so it can be difficult to attribute the observed changes to one specific pathomechanism [29]. The use of IVIM, which offers quantitative perfusion and diffusion evaluation, could potentially be helpful for future studies. 

## 5. Conclusions

Treatment response of targeted therapies such as everolimus might be better depicted by changes in quantitative MRI parameters than conventional size-based criteria. As suggested by the low objective response rates achieved in the RADIANT trials, everolimus only mediates minor tumor shrinkage [4,9]. Our results suggest that the ADC and SI of T1w represent valuable parameters to monitor changes after everolimus treatment and might be able to better differentiate responding from non-responding patients. Interestingly, NELMs of the R group showed a decrease of ADC, which is opposite to the reported changes of DWI after chemotherapy and radiation. However, smaller studies analyzing hypervascular tumors (HCC, RCC) treated by targeted agents also reported decreasing ADC values after therapy. Regarding the major role of imaging response assessment in the development of new treatment strategies, these observations should be further investigated in prospective studies, in a larger multi-center setting.

## Figures and Tables

**Table 1:** Patient characteristics.

Patient Characteristics			
Sex			
Male	11		
Female	6		
Median age, years (range)	68 (27–79)		
Grading			
G1	0		
G2	14 (82%)	median Ki-67 (range)	10 (4–20)
G3	2 (12%)	median Ki-67 (range)	30 (25–40)
n/a	1 (6%)		
median CgA (ng/mL) (range)	1079 (94–29,761)	elevated CgA *, n	13 (76%)
median NSE (ng/mL) (range)	20 (9–87)	elevated NSE *, n	9 (53%)
pNET resected	7 (41%)		
prior medical treatment	14 (82%)		
prior PRRT	8 (47%)		
prior liver-targeted therapy	4 (24%)		

* CgA, ULN 94.0 ng/mL; elevated CgA, greater than 2 × ULN; NSE ULN, 16.3 ng/mL; elevated NSE, greater than 1 × ULN; n, number of patients.

**Table 2:** Cox regression of PFS.

PFS	Univariable			Multivariable		
	HR	95% CI	*p*-Value	HR	95% CI	*p*-Value
Age	0.99	0.96–1.02	0.48			
ki-67	1.03	0.97–1.09	0.37			
Grading	2.25	0.59–8.5	0.23			
Prior systemic treatment	0.95	0.27–3.4	0.94			
Duration of everolimus treatment	0.87	0.74–1.02	0.09	0.88	0.75–1.04	0.14
Elevated CgA *	0.57	0.15–2.2	0.41			
Elevated NSE *	0.54	0.14–2.1	0.37			
% change ADCmin	1.00	0.99–1	0.19			
% change ADCmean	1.01	0.99–1.02	0.19			
DADCmin	0.21	0.06–0.8	0.02	0.30	0.07–1.2	0.09
% change T/L ratio T1	0.99	0.98–1.0	0.10			
% change S/T ratio T2	1.01	0.99–1.03	0.12			

* CgA, ULN 94.0 ng/mL; elevated CgA, greater than 2 × ULN; NSE ULN, 16.3 ng/mL; elevated NSE, greater than 1 × ULN. Significant *p*-values are displayed in bold (according to predefined significance levels).

**Table 3:** Cox regression of OS.

	Univariable		
	HR	95% CI	*p*-Value
Age	1.02	0.97–1.07	0.49
Ki-67	1.00	0.92–1.07	0.87
Grading	0.13	0.13–9.7	0.91
Prior systemic treatment	31.90	0.27–37680	0.34
Duration of everolimus treatment	0.79	0.54–1.16	0.22
Elevated CgA *	1.58	0.19–12.95	0.67
Elevated NSE *	0.77	0.14–4.2	0.76
% change ADCmin	1.00	0.99–1	0.59
% change ADCmean	1.00	0.99–1.01	0.78
DADCmin	0.39	0.07–2.12	0.28
%change T/L ratio T1	0.99	0.97–1.0	0.12
%change S/T ratio T2	1.01	0.99–1.03	0.55

* CgA, ULN 94.0 ng/mL; elevated CgA, greater than 2 × ULN; NSE ULN, 16.3 ng/mL; elevated NSE, greater than 1 × ULN.

## Data Availability

The data presented in this study are available on request from the corresponding author.

## References

[1] Auernhammer C.J., Spitzweg C., Angele M.K., Boeck S., Grossman A., Nölting S., Ilhan H., Knösel T., Mayerle J., Reincke M. (2018). Advanced neuroendocrine tumours of the small intestine and pancreas: Clinical developments, controversies, and future strategies. Lancet Diabetes Endocrinol..

[2] Dasari A., Shen C., Halperin D.M., Zhao B., Zhou S., Xu Y., Shih T., Yao J.C. (2017). Trends in the Incidence, Prevalence, and Survival Outcomes in Patients with Neuroendocrine Tumors in the United States. JAMA Oncol..

[3] Klöppel G., Perren A., Heitz P.U. (2004). The Gastroenteropancreatic Neuroendocrine Cell System and Its Tumors: The WHO Classification. Ann. N. Y. Acad. Sci..

[4] Yao J.C., Pavel M., Lombard-Bohas C., Van Cutsem E., Voi M., Brandt U., He W., Chen D., Capdevila J., De Vries E.G.E. (2016). Everolimus for the Treatment of Advanced Pancreatic Neuroendocrine Tumors: Overall Survival and Circulating Biomarkers from the Randomized, Phase III RADIANT-3 Study. J. Clin. Oncol..

[5] Falconi M., Eriksson B., Kaltsas G., Bartsch D.K., Capdevila J., Caplin M., Kos-Kudla B., Kwekkeboom D., Rindi G., Klöppel G. (2016). ENETS Consensus Guidelines Update for the Management of Patients with Functional Pancreatic Neuroendocrine Tumors and Non-Functional Pancreatic Neuroendocrine Tumors. Neuroendocrinology.

[6] Briest F., Grabowski P. (2014). PI3K-AKT-mTOR-Signaling and beyond: The Complex Network in Gastroenteropancreatic Neuroendocrine Neoplasms. Theranostics.

[7] Pavel M., Öberg K., Falconi M., Krenning E.P., Sundin A., Perren A., Berruti A. (2020). Gastroenteropancreatic neuroendocrine neoplasms: ESMO Clinical Practice Guidelines for diagnosis, treatment and follow-up. Ann. Oncol..

[8] Akirov A., Larouche V., Alshehri S., Asa S.L., Ezzat S. (2019). Treatment Options for Pancreatic Neuroendocrine Tumors. Cancers.

[9] Yao J.C., Shah M.H., Ito T., Bohas C.L., Wolin E.M., Van Cutsem E., Hobday T.J., Okusaka T., Capdevila J., de Vries E.G. (2011). Everolimus for Advanced Pancreatic Neuroendocrine Tumors. N. Engl. J. Med..

[10] Lee L., Ito T., Jensen R.T. (2018). Everolimus in the treatment of neuroendocrine tumors: Efficacy, side-effects, resistance, and factors affecting its place in the treatment sequence. Expert Opin. Pharmacother..

[11] Garcia-Carbonero R., Garcia-Figueiras R., Carmona-Bayonas A., Sevilla I., Teule A., Quindos M., Grande E., Capdevila J., Aller J., Arbizu J. (2015). Imaging approaches to assess the therapeutic response of gastroenteropancreatic neuroendocrine tumors (GEP-NETs): Current perspectives and future trends of an exciting field in development. Cancer Metastasis Rev..

[12] Choi J.-I., Imagawa D.K., Bhosale P., Bhargava P., Tirkes T., Seery T.E., Lall C. (2014). Magnetic resonance imaging following treatment of advanced hepatocellular carcinoma with sorafenib. Clin. Mol. Hepatol..

[13] Krajewski K.M., Nishino M., Franchetti Y., Ramaiya N.H., Abbeele A.D.V.D., Choueiri T.K. (2014). Intraobserver and interobserver variability in computed tomography size and attenuation measurements in patients with renal cell carcinoma receiving antiangiogenic therapy: Implications for alternative response criteria. Cancer.

[14] Solis-Hernandez M.P., Del Valle A.F., Carmona-Bayonas A., Garcia-Carbonero R., Custodio A., Benavent M., Gordoa T.A., Nuñez-Valdovino B., Canovas M.S., Matos I. (2019). Evaluating radiological response in pancreatic neuroendocrine tumours treated with sunitinib: Comparison of Choi versus RECIST criteria (CRIPNET_ GETNE1504 study). Br. J. Cancer.

[15] Ko C.-C., Yeh L.-R., Kuo Y.-T., Chen J.-H. (2021). Imaging biomarkers for evaluating tumor response: RECIST and beyond. Biomark. Res..

[16] Vandecaveye V., Dresen R.C., Pauwels E., Van Binnebeek S., Vanslembrouck R., Baete K., Mottaghy F.M., Clement P.M., Nackaerts K., Van Cutsem E. (2022). Early Whole-Body Diffusion-weighted MRI Helps Predict Long-term Outcome Following Peptide Receptor Radionuclide Therapy for Metastatic Neuroendocrine Tumors. Radiol. Imaging Cancer.

[17] Weikert T., Maas O.C., Haas T., Klarhöfer M., Bremerich J., Forrer F., Sauter A.W., Sommer G. (2019). Early Prediction of Treatment Response of Neuroendocrine Hepatic Metastases after Peptide Receptor Radionuclide Therapy with ^90^Y-DOTATOC Using Diffusion Weighted and Dynamic Contrast-Enhanced MRI. Contrast Media Mol. Imaging.

[18] Wulfert S., Kratochwil C., Choyke P.L., Afshar-Oromieh A., Mier W., Kauczor H.-U., Schenk J.-P., Haberkorn U., Giesel F.L. (2014). Multimodal Imaging for Early Functional Response Assessment of (90)Y-/(177)Lu-DOTATOC Peptide Receptor Targeted Radiotherapy with DW-MRI and (68)Ga-DOTATOC-PET/CT. Mol. Imaging Biol..

[19] Ingenerf M.K., Karim H., Fink N., Ilhan H., Ricke J., Treitl K.-M., Schmid-Tannwald C. (2021). Apparent diffusion coefficients (ADC) in response assessment of transarterial radioembolization (TARE) for liver metastases of neuroendocrine tumors (NET): A feasibility study. Acta Radiol..

[20] Weber M., Kessler L., Schaarschmidt B.M., Fendler W.P., Lahner H., Antoch G., Umutlu L., Herrmann K., Rischpler C. (2020). Treatment-related changes in neuroendocrine tumors as assessed by textural features derived from 68Ga-DOTATOC PET/MRI with simultaneous acquisition of apparent diffusion coefficient. BMC Cancer.

[21] Kukuk G.M., Mürtz P., Träber F., Meyer C., Ullrich J., Gieseke J., Ahmadzadehfar H., Ezziddin S., Schild H.H., Willinek W.A. (2014). Diffusion-weighted imaging with acquisition of three b-values for response evaluation of neuroendocrine liver metastases undergoing selective internal radiotherapy. Eur. Radiol..

[22] Dromain C., Vullierme M., Hicks R.J., Prasad V., O’Toole D., Herder W.W., Pavel M., Faggiano A., Kos-Kudla B., Öberg K. (2022). ENETS standardized (synoptic) reporting for radiological imaging in neuroendocrine tumours. J. Neuroendocr..

[23] Kim J.Y., Hong S.-M., Ro J.Y. (2017). Recent updates on grading and classification of neuroendocrine tumors. Ann. Diagn. Pathol..

[24] Yao J.C., Pavel M., Phan A.T., Kulke M.H., Hoosen S., Peter J.S., Cherfi A., Öberg K.E. (2011). Chromogranin A and Neuron-Specific Enolase as Prognostic Markers in Patients with Advanced pNET Treated with Everolimus. J. Clin. Endocrinol. Metab..

[25] Kurita Y., Kobayashi N., Hara K., Mizuno N., Kuwahara T., Okuno N., Haba S., Tokuhisa M., Hasegawa S., Sato T. (2022). Effectiveness and Prognostic Factors of Everolimus in Patients with Pancreatic Neuroendocrine Neoplasms. Intern. Med..

[26] Rinke A., Auernhammer C.J., Bodei L., Kidd M., Krug S., Lawlor R., Marinoni I., Perren A., Scarpa A., Sorbye H. (2021). Treatment of advanced gastroenteropancreatic neuroendocrine neoplasia, are we on the way to personalised medicine?. Gut.

[27] Padhani A.R., Koh D.-M. (2011). Diffusion MR Imaging for Monitoring of Treatment Response. Magn. Reson. Imaging Clin. N. Am..

[28] Schraml C., Schwenzer N.F., Martirosian P., Bitzer M., Lauer U., Claussen C.D., Horger M. (2009). Diffusion-Weighted MRI of Advanced Hepatocellular Carcinoma During Sorafenib Treatment: Initial Results. Am. J. Roentgenol..

[29] Latour L.L., Svoboda K., Mitra P.P., Sotak C.H. (1994). Time-dependent diffusion of water in a biologicalmodel system. Proc. Natl. Acad. Sci. USA.

[30] García-Figueiras R., Baleato-González S., Padhani A., Luna-Alcalá A., Vallejo-Casas J.A., Sala E., Vilanova J.C., Koh D.-M., Herranz-Carnero M., Vargas H.A. (2019). How clinical imaging can assess cancer biology. Insights Into Imaging.

[31] Jain R., Scarpace L.M., Ellika S., Torcuator R., Schultz L.R., Hearshen D., Mikkelsen T. (2009). Imaging response criteria for recurrent gliomas treated with bevacizumab: Role of diffusion weighted imaging as an imaging biomarker. J. Neuro-Oncol..

[32] Ursprung S., Priest A.N., Zaccagna F., Qian W., Machin A., Stewart G.D., Warren A.Y., Eisen T., Welsh S.J., Gallagher F.A. (2021). Multiparametric MRI for assessment of early response to neoadjuvant sunitinib in renal cell carcinoma. PLoS ONE.

[33] Welsh S.J., Thompson N., Warren A., Priest A.N., Barrett T., Ursprung S., Gallagher F.A., Zaccagna F., Stewart G.D., Fife K.M. (2021). Dynamic biomarker and imaging changes from a phase II study of pre- and post-surgical sunitinib. Br. J. Urol..

[34] Huijts C.M., Santegoets S.J., De Jong T.D., Verheul H., De Gruijl T.D., van der Vliet H. (2017). Immunological effects of everolimus in patients with metastatic renal cell cancer. Int. J. Immunopathol. Pharmacol..

[35] Uutela A., Ovissi A., Hakkarainen A., Ristimäki A., Lundbom N., Kallio R., Soveri L., Salminen T., Ålgars A., Halonen P. (2021). Treatment response of colorectal cancer liver metastases to neoadjuvant or conversion therapy: A prospective multicentre follow-up study using MRI, diffusion-weighted imaging and ^1^H-MR spectroscopy compared with histology (subgroup in the RAXO trial). ESMO Open.

[36] Horger M., Lauer U.M., Schraml C., Berg C.P., Koppenhöfer U., Claussen C.D., Gregor M., Bitzer M. (2009). Early MRI response monitoring of patients with advanced hepatocellular carcinoma under treatment with the multikinase inhibitor sorafenib. BMC Cancer.

[37] Yao J., Shah M., Panneerselvam A., Stergiopoulos S., Chen D., Ito T., Pavel M., Faivre S., Niccoli P., Raoul J. (2012). The Vegf Pathway in Patients with Pancreatic Neuroendocrine Tumors: Efficacy of Everolimus by Baseline Marker Level, and Prognostic and Predictive Effect Analyses from Radiant-3. Ann. Oncol..

[38] Thüring J., Kuhl C.K., Barabasch A., Hitpass L., Bode M., Bünting N., Bruners P., Krämer N.A. (2020). Signal changes in T2-weighted MRI of liver metastases under bevacizumab—A practical imaging biomarker?. PLoS ONE.

